# The association between irregularity in sleep-wake rhythm and CPAP adherence

**DOI:** 10.1038/s44323-024-00001-5

**Published:** 2024-06-19

**Authors:** Aya Honma, Marina Nohara, Sato Honma, Akihiro Homma

**Affiliations:** 1 Department of Otolaryngology-Head and Neck Surgery, Faculty of Medicine and Graduate School of Medicine, Hokkaido University, Sapporo, Hokkaido Japan; 2 Centre for Sleep and Circadian Rhythm Disorders, Sapporo Hanazono Hospital, Sapporo, Hokkaido Japan

**Keywords:** Neuroscience, Diseases

## Abstract

This study aims to evaluate the association between sleep-wake rhythm regularity and continuous positive airway pressure (CPAP) adherence. We retrospectively analyzed sleep-wake rhythms with activity monitoring and CPAP adherence among obstructive sleep apnea (OSA) patients newly diagnosed and introduced to CPAP therapy at the Sapporo Hanazono Hospital from January 2018 to June 2022. Among a total of 45 patients, 10 withdrew from CPAP therapy within a year. Nineteen were classified into the good-adherence and 16 into the poor-adherence group. No significant differences were detected among the groups in apnea-hypopnea index (AHI), sleep efficiency, or subjective sleep quality, but a difference was observed in sleep latency, with the CPAP withdrawal group showing higher variability in sleep onset and lower regularity and/or amplitude in circadian behavior activity rhythm than the good-adherence group. Our results suggest that irregularities, particularly in sleep onset, and damped sleep-wake rhythm can be risk factors for CPAP withdrawal.

## Introduction

Obstructive sleep apnea (OSA) is one of the most common sleep disorders. The pathophysiology of OSA is recurrent apnea or hypopnea during sleep due to upper airway obstruction, leading to a reduction in blood oxygen saturation and an increase in arousal responses. OSA not only reduces subjective sleep quality and causes excessive daytime sleepiness, but is also a risk factor for insulin resistance, hypertension, cardiovascular disease, and even death in severe cases^1–4^. In addition, the risk of motor vehicle accidents was found to be 2 to 3 times higher in OSA patients^5^. Continuous positive airway pressure (CPAP) is the gold standard therapy for moderate to severe OSA^6,7^. CPAP therapy can help decrease blood pressure and prevent cardiovascular events^8–10^. CPAP is also recommended for reducing the risk of motor vehicle accidents^6^. However, CPAP is only successful when used consistently and continuously^11,12^. Thus, poor-CPAP adherence limits its therapeutic effect and, therefore, intervention strategies to improve CPAP adherence are critical to the success of OSA therapy.

In Japan, CPAP therapy is covered by the national health insurance system for patients with moderate or severe OSA, and over 700 thousand OSA patients have been prescribed CPAP^13^. Nevertheless, around 30% of patients quit CPAP within a year^14^. CPAP adherence can be predicted by socioeconomic background, race, marriage, severity of OSA, and the first impressions of CPAP, including CPAP-related adverse events (e.g., nasal obstruction, mouse dryness)^15–18^. In the Sleep Apnea Cardiovascular Endpoints study, Chai-Coetzer et al. reported that CPAP use and adverse events at 1-month predicted long-term CPAP adherence^18^. Approximately two-thirds of CPAP users reported some adverse events, such as nasal symptoms and dry mouth, which contribute to CPAP intolerance^19^. In fact, nocturnal nasal obstruction, presenting in around 30% of OSA patients, affects not only CPAP adherence but also daytime somnolence and quality of life^14,20^. As the initial impressions of CPAP influence intolerance to CPAP therapy, very early interventions are quite important in improving CPAP adherence and managing OSA.

In clinical practice, not a few patients fall asleep before using CPAP due to excessive sleepiness as a symptom of OSA or simply due to inadequate sleep hygiene. Accordingly, they cannot use CPAP on a nightly basis. It is well known that bedtime routines and sleep environment affect sleep quality^21^. Reduced sleep quality, such as increased sleep latency and intermittent awakening may affect tolerance to CPAP therapy^22,23^. Notably, irregularity in sleep-wake rhythm has been reported as a risk factor for various diseases such as cardiovascular disease, hypertension, hyperlipidemia, and diabetes^24–26^. A recent study demonstrated the relationship between sleep irregularity and OSA^27^. An irregular sleep-wake rhythm may also contribute to poor CPAP adherence. Sawyer et al. reported that pre-treatment bedtime variability derived from sleep diaries predicted poor CPAP adherence at 1 month^28^. However, to date, there have been no studies based on objective data on the relationship between sleep-wake rhythm irregularity and long-term CPAP adherence. In this study, we objectively assessed parameters of the sleep-wake rhythm and evaluated their impact on long-term CPAP adherence.

## Results

### Patient profiles and group comparison

**Fig. 1:**
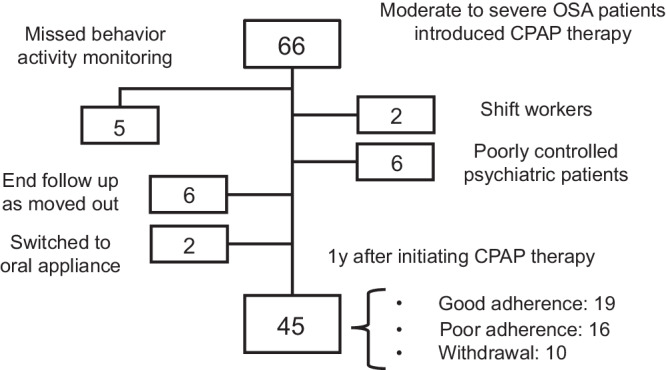
Flowchart of participant selection. A total of 66 OSA participants were referred for the study and 21 were excluded. The remaining 45 patients were categorized into three groups according to their CPAP adherence.

A total of 45 patients (39 males and 6 females) were included in this study. The characteristics of these patients are described in Table 1. We classified CPAP adherence into 3 categories, good adherence: used at least 4 hr/night on ≥70% of the nights per month^29^, poor adherence: used <4 hr/night on >30% of the nights per month, and withdrawal: dropped out within a year. Of the 45 patients included, 19, 16, and 10 were classified into the good-adherence (42.2%), poor-adherence (35.6%), and withdrawal (22.2%) groups, respectively (Fig. 1).

**Table 1:** Characteristics of patients

	All (*n* = 45)	Good adherence (*n* = 19, 42.2%)	Poor adherence (*n* = 16, 35.6%)	Withdrawal (*n* = 10, 22.2%)	*P* value
Sex, male/female	39/6	14/5	15/1	10/0	^–^
Age, y	50.0 (45.5–64.5)	58.0 (46.0–72.0)	48.5 (44.3–52.8)	49.0 (44.0–67.5)	0.0598
BMI, kg/m^2^	25.6 (23.9–30.0)	26.6 (23.2–30.0)	25.5 (24.1–30.6)	25.9 (21.7–30.0)	0.8282
Living alone	17 (37.8)	3 (15.8)	8 (50.0)	6 (60.0)	0.0317
Unemployed	16 (35.6)	10 (52.6)	2 (12.5)	4 (40.0)	0.0609
Hypertension	13 (28.9)	6 (31.6)	3 (18.8)	3 (30.0)	0.6843
Nasal symptom	10 (22.2)	7 (36.8)	2 (12.5)	1 (10.0)	0.1769
Psychiatric comorbidities	22 (48.9)	12 (63.2)	6 (37.5)	4 (40.0)	0.2601
Alcohol	10 (22.2)	4 (25.0)	2 (12.5)	4 (40.0)	0.2998
Smoking	15 (33.3)	5 (26.3)	6 (37.5)	4 (40.0)	0.7974
ESS	10.5 (5.0–16.0)	10.0 (5.0–15.0)	13.0 (6.0–17.5)	7.0 (2.3–15.8)	0.2008
PSQI	9.0 (7.0–12.0)	8.0 (4.0–12.3)	9.0 (8.0–12.5)	11.5 (8.5–12.8)	0.2972
AIS	11.0 (6.0–13.5)	8.5 (5.8–12.3)	12.0 (6.5–13.5)	13.5 (10.0–18.3)	0.0812

Median (IQR) for Kruskal-Wallis post-hoc with Dunn’s multiple comparisons test or number (%) for Fisher’s exact test; *BMI* body mass index, *ESS* Epworth Sleepiness Scale, *PSQI* Pittsburgh Sleep Quality Index, *AIS* Athens Insomnia Scale. Characteristics of Good adherence, Poor adherence, and Withdrawal from CPAP Use ≥4 hr/night at one year of CPAP therapy.

With regard to the withdrawal group, the median (interquartile range: IQR) period until dropout was 2.5 (1.8–4.3) months, with 2 out of 10 patients quitting CPAP therapy within a month. Including these 2 patients, 4 patients dropped out due to exacerbated insomnia symptoms under the CPAP therapy. The other 4 patients were CPAP intolerants and the remaining 2 patients quit CPAP therapy for economic reasons.

Among the three groups, there were no significant differences observed in age, body mass index (BMI), presence of hypertension, nasal symptoms, psychiatric comorbidities, regular alcohol consumption or current smoking habits; however, the differences were found in the rate of living alone (good-adherence: 15.8% vs. poor-adherence: 50% vs. withdrawal: 60%, *n* = 19, 16, 10, respectively, *p* = 0.0317). With regard to self-reported sleep performance from sleep questionnaires, similar scores were observed among the three groups for the Japanese version of the Epworth Sleepiness Scale (ESS)^30,31^, Pittsburgh Sleep Quality Index (PSQI)^32^, and Athens Insomnia Scale (AIS)^33^.

In terms of polysomnography (PSG) data, a significant difference was shown in sleep latency among the 3 groups (good adherence: 7.8 (5.1-15.5) min vs. poor adherence: 4.2 (1.1-9.5) min vs. withdrawal: 3.0 (0.5–7.0) min, *n* = 19, 16, 10, respectively, *p* = 0.0262), while no significant differences were found in other parameters including AHI (Table 2).

**Table 2:** Patient polysomnographic data

	Good adherence (*n* = 19)	Poor adherence (*n* = 16)	Withdrawal (*n* = 10)	*P* value
TST, min	451 (386–515)	471 (433–533)	389 (341–476)	0.1955
Sleep latency, min	7.8 (5.1–15.5)	4.2 (1.1–9.5)	3.0 (0.5–7.0)	0.0262
WASO, min	85.0 (51.7–115.9)	52.4 (26.0–79.4)	98.0 (38.0–145.7)	0.1971
Sleep efficiency, %	83.6 (76.7–88.8)	89.6 (82.2–95.1)	80.9 (64.9–92.2)	0.1092
N3, %TST	2.1 (0.1–8.0)	2.3 (0.3–6.1)	1.8 (0.0–5.7)	0.7121
AHI, events/hr	31.1 (21.5–53.1)	37.6 (24.6–51.6)	35.6 (26.4–58.3)	0.5661

Median (IQR) for Kruskal-Wallis post-hoc with Dunn’s multiple comparisons tests; *TST* total sleep time, *WASO* wake after sleep onset, *AHI* apnea-hypopnea index.

### Sleep-wake rhythm and variability

**Fig. 2:**
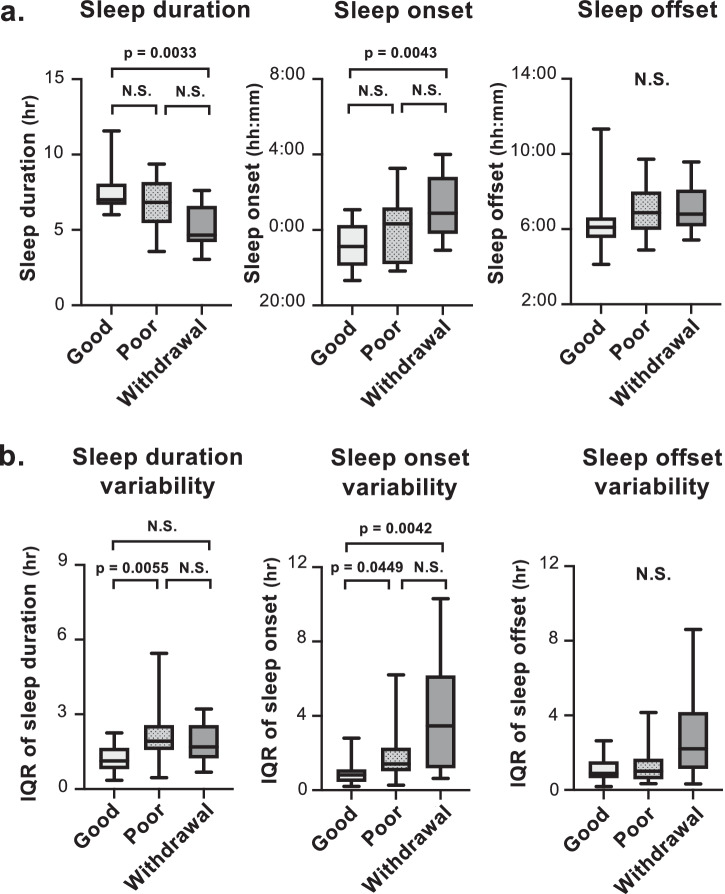
Sleep-wake rhythms and variability. Comparison of sleep-wake rhythms; sleep duration, sleep onset, and sleep offset, among the good-adherence, poor-adherence, and CPAP withdrawal groups (**a**), and their variability (**b**). Median (IQR); Kruskal-Wallis post-hoc with Dunn’s multiple comparisons test.

Prior to PSG, activity monitoring for at least 7 consecutive days was assessed for evaluation of sleep-wake rhythm and behavior rhythmicity. Significant differences were shown in the median (IQR) sleep duration (good-adherence: 7.0 (6.7–8.1) hr vs. poor-adherence: 6.8 (5.5–8.2) hr vs. withdrawal: 4.7 (4.2–6.6) hr, *n* = 19, 16, 10, respectively, *p* = 0.0048) and in sleep onset (23:07 (22:06–0:16) vs. 0:19 (22:12–1:12) vs. 0:53 (23:47–2:49), *n* = 19, 15, 10, respectively, *p* = 0.0044) among the 3 groups, with shorter sleep duration and later sleep onset observed in the withdrawal group compared to in the good-adherence group (post-hoc Dunn’s multiple comparisons test: *p* = 0.0033, *p* = 0.0043, respectively). No significant differences were shown in sleep offset (Fig. 2a).

Variability in sleep-wake rhythms was evaluated using IQR; higher IQR indicated higher variability. Significant differences were shown in the median (IQR) sleep duration variability (good-adherence: 1.1 (0.8–1.7) hr vs. poor-adherence: 1.9 (1.6–2.6) hr vs. withdrawal: 1.7 (1.2–2.6) hr, *n* = 19, 16, 10, respectively, *p* = 0.0043) and in sleep onset variability (0.8 (0.5–1.1) hr vs. 1.4 (1.0–2.3) hr vs. 3.5 (1.2–6.2) hr, *n* = 19, 16, 10, respectively, *p* = 0.0027) were shown among the 3 groups, with higher variability in sleep onset in the poor-adherence and withdrawal groups compared to that in the good-adherence group (post-hoc Dunn’s multiple comparisons test: *p* = 0.0449, 0.0042, respectively). No significant differences were shown in sleep offset variability (Fig. 2b).

### Correlation between CPAP adherence and sleep-wake rhythm

CPAP adherence, as estimated from CPAP usage rate (% > 4 hr/night), showed a positive correlation to sleep duration (*ρ* = 0.4206, *n* = 45, *p* = 0.0040), and a negative correlation to sleep onset (*ρ* = −0.4502, *n* = 44, *p* = 0.0022), but no correlation to sleep offset (Fig. S1a). Negative correlations were also shown between CPAP adherence and variability in sleep duration, sleep onset, and in sleep offset (*ρ* = −0.4536, −0.5475, −0.3484, *n* = 45, 45, 45, *p* = 0.0018, <0.0001 and 0.0190, respectively) (Fig. S1b).

**Fig. 3:**
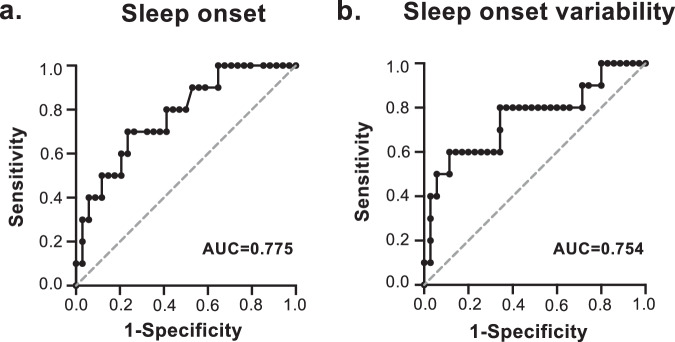
ROC curve of sleep-wake rhythm for predicting CPAP withdrawal. The ROC curves for sleep onset (**a**) and sleep onset variability (**b**) for predicting CPAP withdrawal; cut-off value: 0:26 and 2.5 hr, sensitivity: 70.0% and 60.0%, specificity: 76.5% and 88.6%, respectively.

Receiver Operating Characteristic (ROC) curves demonstrated that a sleep duration of 4.8 hr (Fig. S2a) and sleep onset at 0:26 (Fig. 3a) were the cut-off values for predicting CPAP withdrawal with the area under the ROC curve (AUC): 0.823, 0.775, *n* = 45, 44, *p* = 0.0020 and 0.0088, respectively. The sensitivity and specificity for these cut-off values were 60.0% and 94.3% for sleep duration, and 70.0% and 76.5% for sleep onset, respectively. No statistical significance was detected in the ROC curve for sleep offset. An examination of variability revealed that a sleep onset variability of over 2.5 hr could predict CPAP withdrawal (AUC: 0.754, *n* = 45, *p* = 0.0151) and the sensitivity and specificity of the cut-off value were 60.0% and 88.6%, respectively (Fig. 3b). No statistical significance was shown in the ROC curve for sleep duration variability. Furthermore, among those continuing CPAP therapy, the ROC curve demonstrated that a variability in sleep onset over 0.95 hr predicted poor CPAP adherence with AUC: 0.765, *n* = 35, *p* = 0.0077 (Fig. S2b). The sensitivity and specificity for the cut-off value were 87.5% and 57.9%, respectively.

### Behavior activity rhythm and CPAP adherence

**Fig. 4:**
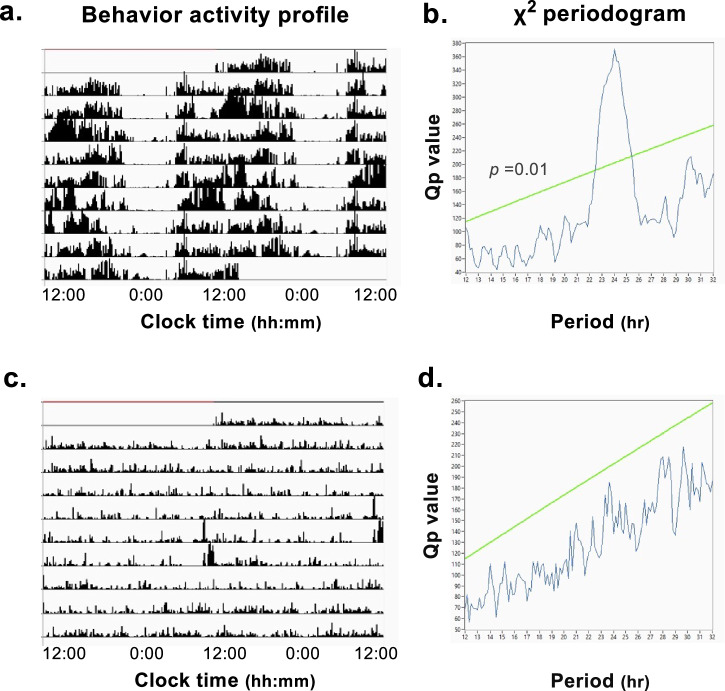
Behavior rhythm analysis. Representative double-plotted behavior rhythms (**a**; rhythmic, **c**; arrhythmic). and their periodograms (**b**; rhythmic, **d**; arrhythmic). The vertical axis of the double-plotted behavior activity profile shows the degree of behavior activity per 10 min.

Two patients showed no periodicity in their behavioral activity rhythm from χ^2^ periodogram analysis: one in the poor-adherence group (6.3%) and the other in the withdrawal group (12.5%). Two patients in the withdrawal group were excluded from the analysis as they only performed activity monitoring at night. Figure 4 shows representative double-plotted behavior activity profiles (a. rhythmic; c. arrhythmic) and periodograms (b. rhythmic; d. arrhythmic).

The χ^2^ periodogram showed significant differences among the 3 groups in the median maximum Qp level, which indicates the regularity and/or amplitude of rhythms (good-adherence: 383 (343–424) vs. poor-adherence: 374 (296–488) vs. withdrawal: 288 (247–363), *n* = 19, 15, 7, *p* = 0.0370). The median of the maximum Qp level in the withdrawal group was lower than that in the good-adherence group (post-hoc Dunn’s multiple comparisons test: *p* = 0.0425), indicating lower regularity and/or amplitude (Fig. S3).

### Other CPAP adherence predictors and sleep-wake rhythm

Sixteen patients living alone showed significantly lower CPAP usage rates (>4 hr/night) compared to patients living with others (30.1 (1.3–46.7) % vs. 86.0 (26.1–98.8) %, *n* = 16, 29, *p* = 0.0024; Fig. S4a). Sleep-wake rhythm variability did not differ significantly between the two groups, but patients living alone showed later sleep onset compared to those living with others (0:25 (23:31–1:11) vs. 23:28 (22:14–0:31), *n* = 16, 28, *p* = 0.0426; Fig. S4b).

Female patients (98.1 (72.7–100) % vs. 44.0 (5.5–94.4) %, *n* = 6, 39, *p* = 0.0146) showed better CPAP adherence than did male patients even though we included only 6 female patients. Age also showed a positive correlation with CPAP usage rate, which means elderly patients had better CPAP adherence (*ρ* = −0.3459, *n* = 45, *p* = 0.0199). There were no correlations observed between CPAP usage rates and BMI nor AHI, and there were no associations with alcohol consumption (14.6 (0.0–86.6) % vs. 63.3 (27.8–96.5) %, *n* = 10, 35, *p* = 0.1622), smoking habit (17.2 (0.0–96.5) % vs. 78.4 (31.3–96.5) %, *n* = 15, 30, *p* = 0.2364), nor comorbidities; hypertension (69.4 (1.3–96.6) % vs. 58.4 (10.2–94.2) %, *n* = 12, 33, *p* = 0.9341), rhinitis (94.3 (33.3–100) % vs. 44.0 (5.6–94.4) %, *n* = 10, 35, *p* = 0.0779), psychiatric comorbidities (77.9 (14.3–100) % vs. 40.9 (5.6–91.9) %, *n* = 22, 23, *p* = 0.1230).

## Discussion

In the present study, we examined the association between sleep-wake rhythm and CPAP adherence in newly diagnosed OSA patients by analyzing their behavior activity monitored before the PSG, and demonstrated that irregularity in sleep onset, short sleep duration, and late sleep onset can be a risk factor for poor CPAP adherence. In the current study, 22.2% of OSA patients quit CPAP therapy within one year, which was a similar rate compared to previous reports in Japan^14,34^. Female and elderly patients showed better CPAP adherence, which was consistent with previous reports^35,36^, but there was no association observed between CPAP adherence and severity of OSA, subjective sleep quality, or daytime sleepiness. Our study demonstrated that living alone could be a predictor of CPAP adherence, which was not unexpected as better CPAP adherence was reported among married patients^16^. More intensive care may be required for patients living alone to improve their CPAP adherence. To our surprise, the presence of a psychiatric comorbidity (e.g., insomnia, depression, or anxiety neurosis) showed no association with CPAP adherence. It is known that OSA patients with insomnia showed poorer CPAP adherence compared to patients with OSA alone^37–39^. In this study, we excluded patients with uncontrolled psychiatric symptoms, and this might explain why no significant differences in CPAP adherence were observed. However, 4 out of 10 patients in the withdrawal group gave up the CPAP therapy due to the worsening of their insomnia. It is understandable that positive pressure from a CPAP mask or a CPAP mask itself may cause difficulties in falling asleep and/or nocturnal awakenings. Alternative treatments are needed when CPAP itself exacerbates patients’ sleep quality, but treatments for insomnia also improve CPAP adherence in patients with OSA and comorbid insomnia symptoms^40^. Health literacy may also affect CPAP adherence^41^. Neither regular alcohol consumption nor current smoking habits were associated with CPAP adherence in this study, although previous studies reported that alcohol and smoking are expected to affect the upper airway collapse in OSA patients^42–44^.

Patients withdrawing from CPAP showed shorter sleep duration and later sleep onset, as well as higher variability in sleep onset compared to those in the good-adherence group, indicating that withdrawing patients were more sleep-deprived, and displayed a stronger eveningness chronotype and a more irregular behavior activity rhythm compared to those with good-adherence. Notably, no significant difference was observed among the three groups in the timing or variability of sleep-offset, which would be due to social schedule and the cause of sleep deprivation in the withdrawal group. When it comes to the poor-adherence group, neither sleep duration nor sleep onset were statistically different from those in the good-adherence group. Only their variability differed. ROC analysis showed the optimal cut-off value of sleep onset variability for CPAP dropout was 2.5 hr, and for poor CPAP adherence, it was 0.95 hr. Therefore, large sleep onset variability can lead to CPAP poor adherence, and if larger than 2.5 hr, it may lead to CPAP dropout. To date, no study has reported on sleep-wake rhythm as a risk factor for CPAP dropout, except a study that demonstrated that the odds of CPAP non-adherence were 3.5 times greater in patients for whom subjectively reported bedtimes varied by >75 min (1.25 hr)^28^. Our results were not inconsistent with those of that report, even though we used sleep onset instead of bedtime. Furthermore, by separately analyzing sleep onset and sleep offset using objective behavior records, we demonstrated that irregularity of sleep onset strongly correlated with CPAP adherence. Sleep-wake rhythms can be desynchronized from circadian rhythms due not only to work schedules but also sleep habits. Misalignment of the circadian rhythm and sleep-wake cycle can reduce the amplitude of circadian rhythms exemplified by a reduction in melatonin levels^45^. Mis-timed sleep in patients with circadian rhythm sleep-wake disorders (e.g., delayed sleep-wake phase disorder and irregular sleep-wake rhythms disorder) impairs sleep quality^46^. The larger day-to-day variance observed in sleep time shown in the withdrawal group may reflect a reduced circadian amplitude (maximum Qp level).

PSG results demonstrated that sleep latency was significantly shorter in the withdrawal group at 3.0 (0.5–7.0) min compared with the good-adherence group at 7.8 (5.1–15.5) min, implying that CPAP withdrawn patients have a sleep debt caused by a lack of sleep. Patients with sleep loss often fall asleep before wearing a CPAP mask, resulting in CPAP non-adherence. As mentioned above, although both the poor-adherence group and withdrawal group showed higher variability in sleep-wake rhythm, only the withdrawal group showed shorter sleep duration, with a possible cause being late sleep onset. Thus, a lack of sleep might give a last push to quitting CPAP.

Our study suggests that sleep-wake rhythm irregularity, particularly sleep onset variability, can be a risk factor against long-term CPAP adherence. Sawyer and colleagues reported in a study using sleep diaries that CPAP non-adherents showed greater variability in bedtime^28^. Given these results, interventions to adjust the circadian rhythms of OSA patients may improve CPAP adherence. A 2020 Cochrane Review reported that behavioral therapies such as cognitive behavioral therapy, motivation, and sleep hygiene instruction were highly recommended to improve CPAP adherence^47^. Their meta-analysis revealed that behavioral therapy increased CPAP usage by 1.31 hr/night, which is clinically meaningful, and may reduce daytime sleepiness (ESS score −2.42 points), even though there were no specific interventions related to sleep-wake rhythm. Although the number of patients in the current study was small, our findings provide evidence to support the importance of sleep-wake rhythm stabilization as an intervention for CPAP adherence.

A limitation of this study was the relatively short observation period for evaluating sleep-wake rhythm. One-week activity records may not be sufficient for sleep-wake rhythm analyses. We ask all patients for at least 2-weeks of activity monitoring in clinical practice, but not a few patients have refused it as activity monitoring is not covered by the national health insurance system. In addition, some patients forgot to wear monitors continuously after the second week. We also did not analyze time in bed, a sleep parameter, as the reliability of the data monitored by an activity tracker is dependent on patient habits, such as reading, checking mail, watching TV and even eating meals in bed. The second limitation was the evaluation methods used for CPAP adherence. We assessed CPAP adherence based on the CPAP usage rate over one month after one year from the initiation of CPAP therapy, and this may not be representative of long-term CPAP adherence, particularly for evaluating good adherence. The third limitation was the ratio of males to females. The majority of patients in this study were males (86.7%), which means that it remains unclear whether the results of this study can be applied to female patients. The fourth limitation was type of CPAP devices and masks. Even though all patients were introduced to automatic CPAP therapy, the type of interface and CPAP device used varied depending on the patient, and this may affect CPAP adherence.

In summary, we examined the association between CPAP adherence and sleep-wake rhythm and demonstrated that an irregular sleep-wake rhythm can be one of the risk factors for early CPAP withdrawal. To improve CPAP adherence, interventions targeting sleep-wake rhythms prior to initiating CPAP therapy are expected to be worthwhile.

## Methods

### Patients

A total of 66 patients with moderate to severe OSA diagnosed by in-laboratory PSG with an AHI of ≥15/hr, who visited the Centre for Sleep and Circadian Rhythm Disorders in Sapporo Hanazono Hospital over the period January 2018 to June 2022 and newly prescribed CPAP were referred retrospectively. Patients who missed behavior activity monitoring prior to the diagnostic PSG (*n* = 5), shift workers (*n* = 2) and patients with poorly controlled psychiatric symptoms; e.g., those undergoing dose adjustment of medication or under inpatient care (*n* = 6), were excluded from the study. Patients who moved out within a year (*n* = 6) and who switched to oral appliance therapy after improving OSA through a decrease in BMI due to diet and exercise therapy (*n* = 2) were also excluded. Finally, 45 patients were included in the study (Fig. 1). The protocol of this investigation was approved by the Sapporo Hanazono Hospital ethics committee (No. 2023-1). All the patients undertaking PSG were asked to provide written informed consent including the analyses of obtained data retrospectively.

### Investigation and assessments

Patients’ information such as comorbidities, employment, drinking, and smoking habits were extracted from the medical interview sheets retrospectively. Briefly, patients filled in the blanks regarding comorbidities, and employment, and made choices from “not drinking alcohol or drinking alcohol (every day/a few times a week / occasionally)” and “non-smoker or smoker” for the drinking and smoking habits. We defined “drinking alcohol every day” as regular alcohol consumption. Daytime sleepiness was assessed using a validated Japanese version of ESS^30,31^. Subjective sleep quality was evaluated by PSQI^32^. Tendency for insomnia was evaluated by AIS^33^. Behavior activity was monitored with an activity tracker J-style AM510N® (Acos CO., LTD., Nagano, Japan) for more than 7 consecutive days. From the amount of behavior activity per 2 min, sleep duration, sleep onset, sleep offset, and 24 hr transition of activity amounts were evaluated. In cases for whom we had more than 7 days of records, the first complete 7 days were selected. Completeness of the activity record is designated as continuous activity records during sleep time for evaluating sleep-wake rhythm. In addition to this, for evaluating 24-hour behavior activity rhythm, we excluded data having missing periods of over an hour during waking hours other than showering or bathing.

The diagnostic overnight full PSG was performed using an Embla N7000® and Embletta MPR® system (Natus, Middleton, WI, USA). All PSG data, sleep stage, and respiratory events were scored manually by independent PSG technicians according to the AASM Manual for the Scoring of Sleep and Associated Events Ver. 2.5 (https://aasm.org/wp-content/uploads/2018/04/Summary-of-Updates-in-v2.5-1.pdf)^48^. For the definition of hypopnea, we followed the recommended criteria of the AASM 2.5.1 with a ≥ 30% reduction in airflow for ≥10 sec associated with arousal or a ≥3% oxygen desaturation, and that of apnea, ≥90% reduction in thermistor signal for ≥10 seconds. All patients were introduced to automatic CPAP therapy under the CPAP adherence guidance by CPAP therapists. CPAP memory data (e.g., CPAP usage rate, days, timing, and median of supplied air pressure and air leak) were confirmed every month and shared with patients. All patients visited the hospital every month or, for those with good adherence, every two months. The benchmark for good adherence was regarded as using CPAP for at least 4 hr/night on ≥70% of the nights over the month. We intervened in patients whose CPAP adherence was poor and fell below this benchmark in accordance with the cause of their difficulty in using CPAP.

CPAP adherence was assessed based on the CPAP usage rate over one month after one year of initiating CPAP therapy. Patients were classified into the following three groups according to CPAP adherence. Good adherence: Patients who used CPAP for at least 4 hr/night on ≥70% of the nights over the month examined, Poor adherence: Patients who used CPAP less than 4 hr/night on >30% of the nights over the month examined, and Withdrawal: Patients who withdrew from CPAP therapy within one year. The CPAP usage rate (% >4 hr/night) of the withdrawal group was evaluated based on the CPAP usage over the last month in which they quit CPAP therapy.

### Data Analysis

For evaluating sleep-wake rhythm, sleep parameters from activity records of seven consecutive days were analyzed using the SleepSignAct® Ver. 2.0 (Kissei Comtec, Nagano, Japan)^49^. Variability in sleep-wake rhythm was evaluated using the IQR of sleep duration, sleep onset, and sleep offset for seven days. The Kruskal-Wallis test post hoc Dunn’s multiple comparisons and Fisher’s exact test were used for analyzing differences among the three groups. Spearman’s rank correlation coefficient was used for the correlation between CPAP usage rate and variability in sleep-wake rhythm. These tests and the ROC curve were analyzed using Prism® Ver. 9.5.1 (GraphPad Software Inc., Boston, MA, USA). To decide the threshold in the ROC analysis, we used the Youden index (*J*), defined as the maximum vertical distance between the ROC curve and the diagonal or chance line. The Youden index (*J*) is calculated as *J* = maximum {sensitivity + specificity −1}^50^.

The rhythmicity of behavior activity was analyzed using ClockLab® (Actimetrics, Lafayette, IN, USA)^51^. Periodicity was determined if the maximum QP level of the χ^2^ periodogram exceeded the significance level of *p* = 0.01. The period with the maximum Qp level was taken as the rhythm period. The regularity and amplitude of behavior activity rhythms were evaluated from the maximum Qp level.

All data were tested by two-tailed and presented as median and IQR (Q1: 25th percentile to Q3: 75th percentile). For tests other than χ^2^ periodogram, P values of less than 0.05 were considered statistically significant and the alpha level was 0.05.

## Supplementary information

Supplementary information

## Data Availability

The datasets generated and/or analyzed during the current study are not publicly available as some data, even when anonymized, could comprise patient privacy, such as complications of mental illness, but are available from the corresponding author upon reasonable request.
