# Supplementary material for: The association between irregularity in sleep-wake rhythm and CPAP adherence
Source: NPJ Biol Timing Sleep. 2024 Jun 19;1:2. doi: 10.1038/s44323-024-00001-5 (PMC12912414; doi:10.1038/s44323-024-00001-5)
Supplement: Supplementary file 1 — Supplementary information [file 44323_2024_1_MOESM1_ESM.docx]

**Supplementary information**

**Figure S1. Correlations between CPAP adherence and sleep-wake rhythms and their variability**

Correlation between CPAP usage rate and sleep-wake rhythm parameters; sleep duration (ρ= 0.4206, n=45, p=0.0040), sleep onset (ρ= -0.4502, n=44, p=0.0022), and sleep offset (ρ= -0.2912, n=44, p=0.0552) (a), and their variabilities (ρ= -0.4536, -0.5475, -0.3484, n=45, 45, 45, p=0.0018, <0.0001 and 0.0190, respectively) (b). ρ; Spearman's rank correlation coefficient.

**Figure S2. ROC curves for sleep-wake rhythm to predict CPAP withdrawal and poor adherence**

The ROC curve for sleep duration showed that the cut-off value for predicting CPAP withdrawal was 4.8 hr, with a sensitivity and specificity of 60.0 % and 94.3 %, respectively (a). The ROC curve for sleep onset variability for predicting CPAP poor adherence showed that a cut-off value of 0.95 hr, with a sensitivity of 87.5 % and a specificity of 57.9 % (b).

**Figure S3. Regularity and/or amplitude of behavior rhythms**

The maximum Qp level in behavior rhythm showed significant differences among the 3 groups (p=0.0370). The withdrawal group showed a lower Qp level compared to the good adherence group, indicating lower regularity and/or amplitude (p=0.0425). Median (IQR); Kruskal-Wallis post-hoc with Dunn‘s multiple comparisons test.

**Figure S4. Lifestyle and CPAP adherence, sleep-wake rhythm**

Patients living alone showed poorer CPAP adherence (a) with later sleep onset (b) compared to those living with others (p=0.0024, 0.0426, respectively). Median (IQR); Mann Whitney test.
